# Risk Factors for Postoperative Urinary Tract Infections in Patients Undergoing Total Joint Arthroplasty

**DOI:** 10.1155/2016/7268985

**Published:** 2016-11-28

**Authors:** Andrew P. Alvarez, Alysen L. Demzik, Hasham M. Alvi, Kevin D. Hardt, David W. Manning

**Affiliations:** Feinberg School of Medicine, Department of Orthopaedic Surgery, Northwestern University, 676 N. St. Clair Street, Suite 1350, Chicago, IL 60611, USA

## Abstract

*Background*. Urinary tract infections (UTIs) are the most common minor complication following total joint arthroplasty (TJA) with incidence as high as 3.26%. Bladder catheterization is routinely used during TJA and the Centers for Medicare and Medicaid Services (CMS) has recently identified hospital-acquired catheter associated UTI as a target for quality improvement. This investigation seeks to identify specific risk factors for UTI in TJA patients.* Methods.* We retrospectively studied patients undergoing TJA for osteoarthritis between 2006 and 2013 in the American College of Surgeon's National Surgical Improvement Program Database (ACS-NSQIP). A univariate analysis screen followed by multivariate logistic regression identified specific patient demographics, comorbidities, preoperative laboratory values, and operative characteristics independently associated with postoperative UTI.* Results.* 1,239 (1.1%) of 115,630 TJA patients we identified experienced a postoperative UTI. The following characteristics are independently associated with postoperative UTI: female sex (OR 2.1, 95% CI 1.6–2.7), chronic steroid use (OR 2.0, 95% CI 1.2–3.2), ages 60–69 (OR 1.5, 95% CI 1.0–2.1), 70–79 (OR 2.0, 95% CI 1.4–2.9), and ≥80 (OR 2.3, 95% CI 1.5–3.6), ASA Classes 3–5 (OR 1.5, 95% CI 1.2–1.9), preoperative creatinine >1.35 (OR 1.8, 95% CI 1.3–2.6), and operation time greater than 130 minutes (OR 1.8, 95% CI 1.3–2.4).* Conclusions.* In this large database query, postoperative UTI occurs in 1.1% of patients following TJA and several variables including female sex, age greater than 60, and chronic steroid use are independent risk factors for occurrence. Practitioners should be aware of populations at greater risk to support efforts to comply with CMS initiated quality improvement.

## 1. Introduction

UTIs represent 13% of all healthcare-associated infections in the United States, resulting in prolonged hospital stays, elevated healthcare expenditures, and increased mortality [1]. It is estimated that the economic burden of healthcare-associated UTIs reaches almost 450 million USD each year, accounting for over 13,000 deaths annually [2, 3].

The incidence of postoperative UTIs specific to TJA has been previously cited as high as 3.26% [4]. Postoperative UTIs have been described as the most common minor systemic postoperative complication following TJA, surpassing deep venous thrombosis, pneumonia, and renal insufficiency [5]. UTIs in the postoperative setting have been linked to significant adverse events such as periprosthetic infection, implant failure, and subsequent revision procedures, resulting in prolonged and costly hospital stays [6–10].

Indwelling bladder catheterization is a well-known risk factor for developing a UTI [11]. To date, there is no widely recognized guideline regarding catheterization in the perioperative setting of TJA, with surgeon preference largely determining bladder management [12, 13]. Routine catheterization has been employed at many surgical centers in order to avoid postoperative urinary retention, something for which patients undergoing TJA are known to be at increased risk, and which itself is associated with UTIs [14, 15].

UTIs are of special interest given the considerable frequency of patient-reported lower urinary tract symptoms in the perioperative setting being as high as 94% [16]. The identification of independently associated risk factors for postoperative UTIs may help orthopaedic surgeons in managing such patients.

A CMS reimbursement policy has recently been proposed in order to increase the focus on quality of care rather than volume [17]. In this proposal, hospitals are not reimbursed for treatment of avoidable complications, such as UTIs, postoperatively following TJA. Avoiding such postoperative adverse events is beneficial not only in terms of patient care but also for surgeon quality metrics and reimbursement. The purpose of this study is to identify the risk factors and to quantify the risk of developing a postoperative UTI following TJA.

## 2. Materials and Methods

This study uses the ACS-NSQIP Database for the years 2006–2013. The ACS-NSQIP Database is a program that collects data preoperatively and up to 30 days postoperatively. This program includes data from over 600 hospitals in 49 of 50 states and in 13 other countries [18]. Each clinical site employs clinical reviewers who collect data through chart review and, if necessary, direct physician or patient contact.

Patients were identified using the CPT codes 27447 and 27130 for total knee and total hip arthroplasty, respectively. Patients were then further selected via ICD-9 code prefix of 715.x to include only those patients with primary diagnosis of osteoarthritis. Patients with concurrent or other procedures performed during the same admission, as well as those with a diagnosis of UTI prior to admission, were excluded. The presence of a UTI was a reported variable for patients in the ACS-NSQIP Database, ascertained via the standardized process as described above.

A univariate analysis (Pearson Chi-Square or Fisher exact test) of all available demographic variables, preoperative laboratory values, comorbidities, and operative characteristics was employed to identify variables significantly associated with a postoperative UTI. A *p* value of 0.05 or less was considered significant. To control for confounders, a multivariate analysis was then utilized. Covariates included in binary logistic regression analysis were those that demonstrated both a significant association with postoperative UTI with a *p* value ≤ 0.2 and had at least 10 occurrences present [19]. Statistical analysis was performed with SPSS version 22 (Chicago, IL).

## 3. Results

1239 (1.1%) of 115,630 total patients who met all inclusion criteria experienced a postoperative UTI. Overall, females experienced postoperative UTIs more frequently than males (1.3% versus 0.8%, *p* < 0.001). The mean age of patients who experienced a postoperative UTI was greater than those who did not (70.89 ± 9.8 versus 66.54 ± 10.3%, *p* < 0.001). Neither race nor BMI (*p* = 0.165 and 0.443, resp.) was risk factors for developing a postoperative UTI (Table 1). Smoking, severe chronic obstructive pulmonary disease, hypertension, and previous percutaneous coronary intervention (PCI)/cardiac surgery, as well as previous TIA or stroke were significantly associated with postoperative UTIs (all *p* < 0.05); however, congestive heart failure and disseminated cancer were not.

Patients with an ASA classes 3–5 were more likely to experience a postoperative UTI as compared to those with ASA classes 1-2 (1.4% versus 0.8%, *p* < 0.001). Additionally, patients undergoing general anesthesia were more likely to develop a postoperative UTI compared to those undergoing spinal or other (including regional and local) anesthesia (1.1% versus 1.0% versus 0.8%, respectively, *p* = 0.022) (Table 2). Evaluation of preoperative laboratory values revealed elevated creatinine and were associated with postoperative UTIs (*p* < 0.05); however, platelets and hematocrit were not (Table 2).

A multivariate binary logistic model was able to identify which variables were independently associated with postoperative UTIs, adjusting for confounding variables (Table 3). Increasing age was identified as the strongest variable in developing a postoperative UTI: 60–69 (OR, 1.5; 95% CI, 1.0–2.1), 70–79 (OR, 2.0; 95% CI, 1.4–2.9), and ≥80 (OR, 2.3; 95% CI, 1.5–3.6) (Table 3). Female sex was also strongly associated with increased odds of postoperative UTI in our multivariate analysis (OR, 2.1; 95% CI, 1.6–2.7), as were steroid use (OR, 2.0; 95% CI, 1.2–3.2), operative time greater than 130 minutes (overall mean + 1 SD) (OR, 1.8; 95% CI, 1.3–2.4), and preoperative creatinine greater than 1.35 (overall mean + 1 SD) (OR, 1.8; 95% CI, 1.3–2.6).

## 4. Discussion

With recent nationwide quality initiatives to minimize healthcare-associated infections, the ability to identify and manage patients at the greatest risk of postoperative UTIs has become an increasingly important topic in the orthopaedic arena. While many of these quality improvement initiatives have helped to limit bladder catheterization and thus prevent UTIs, it is clear that UTI in the postoperative TJA setting is not uncommon.

Considerable research has been conducted regarding nosocomial UTIs in general; however, research pertaining to UTIs strictly in TJA patients is sparse [11, 14, 15, 20]. Moreover, previously established risk factors for any nosocomial UTI, including female sex, diabetes mellitus, and elevated creatinine, were consistent with the findings in our study [11]. Whether the additional risk factors identified in our study are unique to the TJA population or broadly applicable to other patient groups, cannot be definitively concluded at this time.

With regards to postsurgical patients in other surgical specialties, research indicates that the appreciable incidence of postoperative UTIs is not exclusive to orthopaedic surgery [20]. One study investigating the incidence of postoperative UTIs following major surgeries in various specialties revealed that the rates are indeed similar across multiple surgical services: 30-day postoperative UTI rate for coronary artery bypass, vascular, colorectal, and TJA surgeries were 3.3, 3.4, 4.0, and 3.4%, respectively [20].

It is recognized that our study is limited by the absence of patient bladder catheterization data (including presence of indwelling catheter and duration) within the ACS-NSQIP Database, which may have impacted results. For example, it can be hypothesized that patient populations with characteristics we found to be significantly associated with postoperative UTIs, such as advanced age or ASA status 3–5, were more likely to be catheterized and thus at increased risk for UTI. Further studies with strict data collection would be able to provide more clarification.

Another potential limitation of this study includes its retrospective nature, as well as the intrinsic variability in data collection from multiple sites. However, the ACS-NSQIP program has implemented extensive training and auditing procedures for each participating hospital to ensure the collection of reliable data and a high level of verified interrater reliability [21]. Various analyses have demonstrated that, across most hospitals and models, the reliability of the ACS-NSQIP data is reasonable for assessing surgical quality [22].

Our study has identified multiple characteristics independently associated with postoperative UTIs following TJA, which may be useful to clinicians in identifying at-risk patients. While this information alone may have the potential to improve the quality of patient care, at this time, the clinical utility of these risk factors is unproven. Further research such as a prospective study stratifying patients into risk groups to guide postoperative management or perioperative catheterization may be employed to establish practical utility.

## Figures and Tables

**Table 1:** Preoperative characteristics.

Characteristic	No UTI		UTI		*p* value
Number of patients	114391	98.9%	1239	1.1%	
Age (years) (mean, SD)^*∗*^	66.54, 10.3		70.89, 9.8		<0.001
<59	28165	99.4%	160	0.6%	
60–69	40511	99.1%	353	0.9%	
70–79	32296	98.6%	453	1.4%	
80+	12717	98.0%	258	2.0%	
BMI (mean, SD)	31.8, 7.1		32.1, 7.4		0.443
Underweight	681	98.6%	10	1.4%	
Normal	15569	98.9%	166	1.1%	
Overweight	34871	99.0%	349	1.0%	
Obese (Class 1)	30823	98.9%	348	1.1%	
Obese (Class 2)	18441	98.9%	201	1.1%	
Obese (Class 3)	13787	98.8%	162	1.2%	
Sex^*∗*^					<0.001
Male	45263	99.2%	343	0.8%	
Female	68968	98.7%	895	1.3%	
Race					0.165
White	91973	99.0%	967	1.0%	
Black	7190	98.7%	92	1.3%	
Asian	2024	99.2%	16	0.8%	
Other/unknown	4533	98.8%	53	1.2%	
Current smoker^*∗*^	11107	99.3%	82	0.7%	<0.001
Steroid use^*∗*^	3229	97.9%	68	2.1%	<0.001
Comorbidities					
Diabetes mellitus^*∗*^					<0.001
Non-insulin dependent	13465	98.7%	171	1.3%	
Insulin-dependent	4122	98.2%	74	1.8%	
Disseminated cancer	143	97.3%	4	2.7%	0.074
Dyspnea^*∗*^	7528	98.5%	115	1.5%	<0.001
COPD^*∗*^	4163	98.4%	68	1.6%	0.001
Hypertension^*∗*^	72760	98.8%	916	1.2%	<0.001
Open wound/wound infection^*∗*^					0.002
Congestive heart failure < 30 days	267	98.2%	5	1.8%	0.223
Bleeding disorders^*∗*^	2952	98.2%	53	1.8%	<0.001
Previous PCI/cardiac surgery^*∗*^	3311	98.1%	64	1.9%	0.006
Previous TIA/stroke^*∗*^	1793	98.1%	35	1.9%	0.04
ASA class^*∗*^					<0.001
1, 2	63731	99.2%	513	0.8%	
3, 4, 5	50541	98.6%	723	1.4%	
Functional status^*∗*^					<0.001
Dependent	2616	98.2%	49	1.8%	
Independent	111250	98.9%	1185	1.1%	

*∗* denotes significance.

**Table 2:** Operative and lab characteristics.

Characteristic	No UTI		UTI		*p* value
Total operative time (mins) (mean, SD)	92.63, 37.2		95.21, 45.5		0.128
<130.0	100143	98.9%	1067	1.1%	
>130.0	14234	98.8%	172	1.2%	
Anesthesia type^*∗*^					0.022
General	61043	98.9%	710	1.1%	
Spinal	43710	99.0%	452	1.0%	
Other (including regional and local)	5776	99.2%	48	0.8%	
Creatinine > mean + 1 SD (1.35)^*∗*^	6180	98.1%	121	1.9%	<0.001
Platelets < 50	195	99.0%	2	1.0%	0.934
Hematocrit values < 21	184	98.9%	2	1.1%	0.999
INR > 2^*∗*^	751	98.2%	14	1.8%	0.047

*∗* denotes significance.

**Table 3:** Independent risk factors for postop UTI as defined by multivariate logistic regression.

Variable	OR (95% CI)
Sex (female)^*∗*^	2.1 (1.6–2.7)
Diabetes	
Non-insulin dependent	1.2 (0.9–1.7)
Insulin dependent	1.6 (0.9–2.5)
Smoker	1.0 (0.6–1.5)
Dyspnea	0.9 (0.6–1.4)
Functional status (dependent)	0.9 (0.5–1.6)
COPD	1.2 (0.7–2.0)
Hypertension	0.9 (0.7–1.2)
Steroid use^*∗*^	2.0 (1.2–3.2)
Bleeding disorder	1.3 (0.8–2.2)
Age	
60–69^*∗*^	1.5 (1.0–2.1)
70–79^*∗*^	2.0 (1.4–2.9)
≥80^*∗*^	2.3 (1.5–3.6)
History of PCI/cardiac surgery	1.3 (0.9–1.8)
History of TIA/stroke	1.1 (0.7–1.7)
ASA classes 3–5^*∗*^	1.5 (1.2–1.9)
Preop Cr > 1.35^*∗*^	1.8 (1.3–2.6)
Preop INR > 2	0.6 (0.2–2.0)
Operation time > mean + 1 SD (130 mins.)^*∗*^	1.8 (1.3–2.4)
Anesthesia type	
Spinal	0.9 (0.7–1.2)
Other	0.7 (0.4–1.3)

*∗* denotes significance.

## Abbreviations

UTI: Urinary tract infection
TJA: Total joint arthroplasty
CMS: Centers for Medicare and Medicaid Services
ACS-NSQIP: American College of Surgeon's National Surgical Improvement Program
CPT: Current Procedural Terminology
ICD-9: International Classification of Diseases, 9th Revision
INR: International normalized ratio.
